# Pelvic obliquity, trunk control, and motor function: an exploratory study in a non-ambulatory Duchenne muscular dystrophy cohort

**DOI:** 10.1590/1806-9282.20241109

**Published:** 2024-12-02

**Authors:** Fatma Uğur, İpek Gürbüz, Özlem Yayıcı Köken, Ayşegül Neşe Çıtak Kurt, Öznur Yılmaz

**Affiliations:** 1 Karamanoglu Mehmetbey University, Faculty of Health Sciences – Karaman, Turkey.; 2 Hacettepe University, Faculty of Physical Therapy and Rehabilitation – Ankara, Turkey.; 3 Akdeniz University, Faculty of Medicine, Department of Pediatric Neurology – Antalya, Turkey.; 4 Ankara Yıldırım Beyazıt University, Ankara City Hospital, Department of Pediatric Neurology – Ankara, Turkey.

**Keywords:** Wheelchairs, Duchenne muscular dystrophy, Scoliosis, Pelvis

## Abstract

**OBJECTIVE:**

The aim of this study was to investigate the relationship between pelvic obliquity and trunk control, spinal deformity, upper extremity functional performance, and motor function in patients with non-ambulatory Duchenne muscular dystrophy.

**METHODS:**

This cross-sectional study included 21 patients with Duchenne muscular dystrophy aged 8–18 years. In the study, participants’ upper extremity functional levels, pelvic obliquity degrees, spinal deformity, trunk control, upper extremity performance, and motor functions were evaluated using various tools such as the Brooke Upper Extremity Functional Scale, baseline scoliometer, spinal mouse, Trunk Control Measurement Scale, Upper Extremity Performance Scale, and Egen Klassifikation Scale Version 2, respectively.

**RESULTS:**

The study found a strong correlation between pelvic obliquity and frontal spine deformity (p<0.01), as well as moderate relationships between pelvic obliquity, motor function, wheelchair usage duration, and knee flexion contractures (p<0.05).

**CONCLUSION:**

The study found that pelvic obliquity may lead to spinal health deterioration and motor function limitations in non-ambulatory patients with Duchenne muscular dystrophy, especially those with lower extremity joint contractures and long-term wheelchair use.

## INTRODUCTION

Duchenne muscular dystrophy (DMD) is a prevalent childhood neuromuscular disease (NMD) affecting 1 in every 3,800–6,300 males with X-linked genetic inheritance. It causes progressive muscle weakness in the proximal extremities and trunk, with secondary effects including muscle shortness, joint contracture, scoliosis, kyphosis, increased lumbar lordosis, and pelvic obliquity (PO)^1,2^.

PO is a pelvic asymmetry in the frontal plane, resulting from spinal deformity, muscle weakness, hip contractures, lower extremity inequality, or a combination of these^3^. PO may result from a suprapelvic, intrapelvic, or infrapelvic problem. The suprapelvic problem is the asymmetry of the pelvis that is secondary to scoliosis. Intrapelvic problems include deformities of the sacro-pelvic unit. Infrapelvic problems, however, indicate deformities around the hip joint^3,4^. In DMD, “lower-origin (infrapelvic) PO” develops secondary to hip contractures, whereas “upper-origin (suprapelvic) PO” develops secondary to scoliosis^5^.

The literature contains studies that investigate the relationship between the trunk, which is the key point of the kinematic chain in DMD, and upper extremity function^6,7^. However, no study was detected in the literature investigating the relationship between PO, trunk control, and motor function in non-ambulatory patients with DMD. The purpose of this study was to investigate the relationship between PO and trunk control, spinal deformity in the frontal and sagittal planes, upper extremity functional performance, and motor function in non-ambulatory patients with DMD.

## METHODS

The study was conducted at X University’s Faculty of Physical Therapy and Rehabilitation, Pediatric Neuromuscular Diseases Unit, using a cross-sectional observational methodology. It received ethical approval from the X City Hospital Clinical Research Ethics Committee (number E2-21-62) and adhered to the Helsinki Declaration standards. DMD participants and their parents signed a consent form after receiving the information.

### Participants

The study involved participants diagnosed with DMD aged 8–18 years who could not walk for at least 6 months, could sit for 30 min without back support, followed physiotherapist instructions, and had no other neurological or orthopedic diagnoses. Participants with injuries or surgeries in the last 6 months and severe lower or upper extremity contractures were excluded from the study.

### Assessments

The demographic data of participants, such as age (years), height (cm), weight (kg), body mass index (kg/m^2^), steroid usage, and age of onset of wheelchair use, were recorded.

Participants with DMD had their upper extremity functional levels assessed using the Brooke Upper Extremity Functional Scale (BUEFS). BUEFS was developed specifically for use in DMD. The total score ranges from 1 to 6 and is scored as “1” when the patient starts from the sides and folds his arms above his head and “6” when he cannot even bring his hands to his mouth and cannot use them functionally^8^.

Muscle shortness around the hip joint muscles [the lumbar extensor, quadriceps, tensor fasciae latae (TFL), hip flexor, hamstring, and gastrocnemius] was assessed^9^.

The HF, KF, and APF contractures were measured passively with a goniometer and recorded in “degrees”^10^.

The study evaluated PO in patients with DMD using the “Baseline Scoliometer,” a non-invasive device used to measure trunk asymmetry in scoliosis. The scoliometer showed excellent intraobserver and interobserver reliability in determining PO in patients with spinal muscular atrophy (SMA)^11^. PO was evaluated in a sitting position with the participants’ lower extremities supported, with the physiotherapist marking the spina iliaca posterior superior (SIPS) and measuring the asymmetry in “degrees” between the two points.

Spinal status, spine angle, and shape in the frontal and sagittal planes were assessed using the spinal mouse (SM), a non-invasive measuring device. SM can determine thoracic kyphosis and lumbar lordosis, as well as the angle, direction, and location of the spine’s lateral curvature. Spinal curvatures can be detected early with SM, allowing for early intervention against curvature progression. SM is safe for clinical research and patient follow-up in evaluating structural deformities like scoliosis^12^. Participants with DMD were measured while sitting with their lower extremities supported. Measurements were taken on the skin’s surface at a constant speed and pressure. The frontal spine curvature and thoracic kyphosis angle were determined in “degrees.”

The “Trunk Control Measurement Scale (TCMS)” was used to assess trunk control levels in patients with DMD. The scale, consisting of 15 items, evaluates static and dynamic sitting balance, as well as trunk compensation during limb movements. A higher score indicates (total score: 0–58 points) better control, and the TCMS’s validity and reliability were found to be good in neuromotor impairment patients^13^.

In participants with DMD, the “The Performance of Upper Limb Scale (PUL)” was used to evaluate upper extremity function and movement quality^7^, created by Mayhew et al.^14^. The scale consists of 23 items. Each item is scored between “0 to 1 point” and “0 to 6 points” according to the scoring system. The total score ranges from 0 to 74 points. Higher scores indicate better upper extremity functional performance.

“The Egen Klassifikation Scale Version 2 (EK2)” was used to assess the motor functional abilities of non-ambulatory patients with DMD. Alemdaroğlu et al. conducted a 2014 study on the validity and reliability of the Turkish version of EK2 for non-ambulatory DMD and SMA patients^15^. EK2 assesses daily functional activities like wheelchair use, transfer ability, arm function, feeding, turning in bed, coughing, and speaking. The items are scored between 0 and 3, with a total score ranging from 0 to 51 points. Low scores indicate higher motor function.

### Statistical analysis

The IBM SPSS Statistics 23 analysis program was used for statistical analysis. The quantitative data from descriptive statistics, including demographic and physical characteristics of participants with DMD, were defined by the minimum (min.), maximum (max.), mean (X), and standard deviation (SD), while the qualitative data were defined as numbers (n) and percentages (%). The data’s conformity to the normal distribution was assessed using the Shapiro-Wilk test, revealing non-compliance with the normal distribution assumption. The correlation between variables was determined using Spearman’s correlation coefficient. The coefficient value (rs) of 0.00–0.10 showed insignificant correlation, 0.10–0.39 weak correlation, 0.40–0.69 moderate correlation, 0.70–0.89 strong correlation, and 0.90–1.00 very strong correlation^16^. The statistical significance level was accepted as p<0.05.

## RESULTS

The study group consisted of 21 DMD patients with a mean age of 12.5±2.3 years, a mean weight of 44.3±9.5 kg, a mean height of 147.2±10 cm, and a mean BMI of 20.3±3 kg/m^2^. In this study, a large effect size (r=0.78) and 89% power were achieved with a 95% confidence interval, according to the G-POWER power analysis program. According to BUEFS, 38.10, 14.29, 14.29, 9.52, 9.52, 9.52, and 23.81% of the participants with DMD had a Brooke 1, 2, 3, 4, and 5 upper extremity functional level, respectively. Nearly all participants exhibited shortness in the gastrocnemius and hamstring muscles, while most also displayed shortness in the hip flexor and TFL muscles. Table 1 provides information on muscle shortness and joint contractures. All participants had ankle plantarflexion (APF), and most of them had knee flexion (KF) contractures. There was a moderate correlation between PO and KF contractures. With the exception of muscle shortness and joint contracture, Table 2 presents the findings of all evaluation parameters, and Table 3 presents the relationships between PO and evaluation parameters. There was a strong correlation between PO and frontal spine deformity (p<0.01), and there were also moderate correlations between PO, EK2, and wheelchair usage duration (Table 3) (p<0.05).

**Table 1:** Muscle shortness, joint contractures, and relationships between the degree of pelvic obliquity of non-ambulatory patients with Duchenne muscular dystrophy (n=21).

Muscle shortness	n (%)	Joint Contractures	X±SD	r^a^
Gastrocnemius		Ankle plantarflexion (APF)		
Right	21 (100)	Right	58.57±18.82	0.39
Left	21 (100)	Left	57.33±18.25	0.4
Hamstring		Knee flexion (KF)		
Right	21 (100)	Right	34.43±29.18	0.52*
Left	20 (95.2)	Left	33.67±29.22	0.47*
Hip flexor		Hip flexion (HF)		
Right	18 (85.7)	Right	1.43±6.55	-0.09
Left	18 (85.7)	Left	1.67±7.64	-0.09
TFL				
Right	13 (61.9)			
Left	14 (66.7)			
Quadriceps				
Right	9 (42.9)			
Left	8 (38.1)			
Lumbar extensor				
Yes	6 (28.6)			

*Statistically significant relationship (p<0.05); TFL: tensor fasciae latae;

^a^relationships with pelvic obliquity degree and joint contractures; X±SD: mean±standard deviation; r: correlation coefficients.

**Table 2:** Values of pelvic obliquity and other outcome measures in non-ambulatory patients with Duchenne muscular dystrophy (n=21).

Outcome Measures	X±SD	Min–Max
PO (degree)	6.14±6.63	0–30
TCMS (point)	26.05±17.48	2–52
PUL total (point)	52.05±15.47	23–70
PUL high level (point)	4.33±4.58	0–12
PUL mid-level (point)	25±10.77	3–34
PUL distal level (point)	22.71±1.19	20–24
SM thoracic kyphosis (degree)	29.55±9.73	14.25–54.33
SM frontal curvature (degree)	14.01±14.45	1.75±61.66
EK-2 (point)	13.67±7.62	4–28
Wheelchair usage time (year)	3±2.05	1–8
Age at onset of wheelchair use (year)	9.52±1.50	6–12
Steroid duration (year)	6.31±2.64	1.5–11
Trunk length (cm)	40.49±3.40	34.45–45.35

PO: pelvic obliquity; TCMS: Trunk Control Measurement Scale; PUL: the performance of upper limb; SM: spinal mouse; EK-2: Egen Classification Scale 2; X±SD: mean±standard deviation; min: minimum; max: maximum.

**Table 3:** Relationships between the degree of pelvic obliquity and other outcome measures in non-ambulatory patients with Duchenne muscular dystrophy (n=21).

Outcome measures	1	2	3	4	5	6	7	8
1. PO	–							
2. TCMS	-0.32	–						
3. PUL total	-0.41	0.82**	–					
4. SM thoracic kyphosis	-0.15	0.15	0.22	–				
5. SM frontal curvature	0.78**	-0.23	-0.28	-0.10	–			
6. EK-2	0.52*	-0.86**	-0.78**	-0.25	0.33	–		
7. Wheelchair usage duration	0.50*	-0.64**	-0.69**	-0.30	0.21	0.73**	–	
8. Steroid usage duration	0.12	-0.29	-0.44	0.08	0.02	0.26	0.45	–
9. Trunk length	0.43	-0.19	-0.23	-0.09	0.29	0.32	0.41	0.62**

*Statistically significant relationship (p<0.05);

**Statistically significant relationship (p<0.01); PO: pelvic obliquity; TCMS: Trunk Control Measurement Scale; PUL: the performance of upper limb; SM: spinal mouse; EK-2: Egen Classification Scale.

## DISCUSSION

The study found that PO is associated with frontal spine deformity, motor function, wheelchair usage duration, and KF contractures in non-ambulatory patients with DMD.

Thoracolumbar scoliosis-PO-hip subluxation/dislocation is termed the “*Triad of Neuromuscular Deformities*” or “*The Triadic Complex*.” In DMD, scoliosis is widely believed to cause pelvic tilt or PO^17^. Dubious Sets concept identifies four distinct pelvic slopes^3^. Pelvic asymmetry is classified as “*Type 1 pelvic tilt*” in patients with DMD. In a study of 116 NMS patients (including those with DMD), PO was found in 98%^17^. Choi et al.^18^ found a strong relationship between Cobb angle and PO in patients with DMD, suggesting PO occurs only in those with scoliosis. Patel et al.^17^ found that in non-ambulatory patients with NMD, spinal deformity precedes hip deformity. However, Chan et al.^19^ reported a notable (35%) incidence of hip subluxation and dislocation in DMD, with a positive correlation to PO. The study found that PO is linked to spine frontal curvature, but it is unclear if PO directly influences scoliosis or vice versa, despite evidence of a relationship^2,19^.

Functional capacity declines as DMD progresses^20^. Connolly et al.^21^ found that in non-ambulatory DMD patients, EK Scale scores significantly decreased over 2 years, indicating a sensitive measure of change. This study revealed a linear relationship between EK2 scores and PO degree, suggesting precision motor function measurement reflects increased PO degree in non-ambulatory DMD patients.

In DMD, muscle weakness and secondary musculoskeletal problems worsen over time, leading to wheelchair dependence^22^. Shapiro et al.^23^ reported that prolonged wheelchair use (median 36 months) in DMD patients correlates with the severity of scoliosis, irrespective of age. In our study, a moderate association was found between moderate PO^17^ and a median wheelchair duration of 3 years. The convergence of findings suggests that a 3-year wheelchair duration may signal significant spinal deformity in DMD. In future longitudinal studies, it should be investigated whether wheelchair usage has a cut-off value to determine spinal deformities, which may eventually guide the rehabilitation process.

In children with DMD, the frequency and severity of lower extremity joint contractures increase rapidly after ambulatory function loss^10^. Willcocks et al.^24^ emphasized that APF, HF, and KF contractures were observed before loss of ambulatory function in patients with DMD, respectively. In our study, participants with DMD were categorized as having severe APF (59°), moderate KF (34°), and mild HF (1°) contractures. The reason for KF<APF in both studies may be that APF contracture is compensatory and helps knee stability. In our study, no correlation was observed between severe APF contracture and PO, whereas a moderate correlation was observed between moderate KF contracture and PO. A possible explanation for this may be that the knee joint is located closer to the spine than the ankle joint. Our findings point to the predictive importance of KF contracture for the clinical management of PO.

The study found moderate-strong relationships between TCMS-PUL total, TCMS-EK2, and TCMS-wheelchair usage duration, but no correlation between PO and TCMS or PUL total scores. Current functional scales like PUL are used to assess upper extremity limitations in DMD but may not capture the full range of patients due to floor and ceiling effects^14^. The lack of correlation between PO and upper extremity functions could be attributed to the ceiling effect of PUL, which is unable to detect upper extremity disorders at the earliest stage^25^. The absence of a relationship between trunk control and upper extremity functions may stem from the relatively low PO degree in patients with DMD. Existing literature suggests an indirect relationship between pelvis, trunk control, and upper extremity performance^6,7,17^.

The study’s cross-sectional design did not explore longitudinal changes in PO grades in non-ambulatory patients with DMD. Future research should monitor PO grades longitudinally to understand their role in scoliosis development. The evaluation of spinal deformities in DMD usually happens after ambulation loss, which restricts our comprehension of deformities during the late ambulatory period.

## CONCLUSION

The study suggests that PO can lead to spinal health deterioration and motor function limitations in non-ambulatory patients with DMD, especially those with lower extremity joint contractures and who use a wheelchair for extended periods.
